# Characterizing the Monomer–Dimer Equilibrium of UbcH8/Ube2L6: A Combined SAXS and NMR Study

**DOI:** 10.1021/acsomega.4c03610

**Published:** 2024-09-09

**Authors:** Kerem Kahraman, Scott A. Robson, Oktay Göcenler, Cansu M. Yenici, Cansu D. Tozkoparan Ceylan, Jennifer M. Klein, Volker Dötsch, Emine Sonay Elgin, Arthur L. Haas, Joshua J. Ziarek, Çağdaş Dağ

**Affiliations:** † Nanofabrication and Nanocharacterization Center for Scientific and Technological Advanced Research (n^2^STAR), Koç University, İstanbul 34450, Turkey; ‡ Department of Pharmacology, Feinberg School of Medicine, Northwestern University, 320 East Superior Avenue, Chicago, Illinois 460611, United States; § Department of Biochemistry and Molecular Biology, LSUHSC-School of Medicine, 1901 Perdido Street, New Orleans, Louisiana 70112, United States; ∥ Centre for Biomolecular Magnetic Resonance, Institute for Biophysical Chemistry, Goethe-University of Frankfurt/Main, Frankfurt 60439, Germany; ⊥ College of Sciences, Department of Chemistry, Muğla Sıtkı Koçman University, Muğla 48000, Turkey; # Koç University Isbank Center for Infectious Diseases (KUISCID), Koç University, Istanbul 34450, Turkey

## Abstract

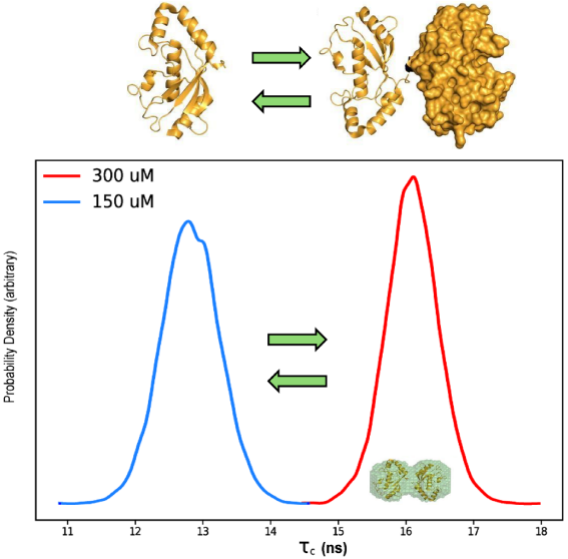

Interferon-stimulated gene-15 (ISG15) is an interferon-induced protein with two ubiquitin-like (Ubl) domains linked by a short peptide chain and is a conjugated protein of the ISGylation system. Similar to ubiquitin and other Ubls, ISG15 is ligated to its target proteins through a series of E1, E2, and E3 enzymes known as Uba7, Ube2L6/UbcH8, and HERC5, respectively. Ube2L6/UbcH8 plays a central role in ISGylation, underscoring it as an important drug target for boosting innate antiviral immunity. Depending on the type of conjugated protein and the ultimate target protein, E2 enzymes have been shown to function as monomers, dimers, or both. UbcH8 has been crystallized in both monomeric and dimeric forms, but its functional state remains unclear. Here, we used a combined approach of small-angle X-ray scattering (SAXS) and nuclear magnetic resonance (NMR) spectroscopy to characterize UbcH8’s oligomeric state in solution. SAXS revealed a dimeric UbcH8 structure that could be dissociated when fused N-terminally to glutathione S-transferase. NMR spectroscopy validated the presence of a concentration-dependent monomer–dimer equilibrium and suggested a back-side dimerization interface. Chemical shift perturbation and peak intensity analysis further suggest dimer-induced conformational dynamics at the E1 and E3 interfaces, providing hypotheses for the protein’s functional mechanisms. Our study highlights the power of combining NMR and SAXS techniques to provide structural information about proteins in solution.

## Introduction

Interferon-stimulated gene 15 (ISG15), also known as hUCRP or IP17, is a 15 kDa ubiquitin-like, type I interferon (IFN)-inducible protein.^1^ ISGylation is a ubiquitin-like (Ubl) post-translational modification (PTM) that involves the covalent attachment of ISG15 to target proteins.^2^ Similar to other Ubls, ISGylation plays important roles in various cellular processes, such as innate antiviral immunity, protein degradation, and signal transduction.^3^ Free, unconjugated ISG15 also serves immunoregulatory functions as a cytoplasmic and secreted signaling protein in eukaryotic organisms.^4^ Inherited ISG15 deficiency dramatically reduces the innate immune system’s ability to fight viruses in mice, yet it only appears to cause immunoregulatory issues against mycobacterial, not viral diseases, in humans.^3^ Thus, the role of ISG15 in human viral pathogenesis is not clearly understood.

The ISGylation cascade requires the sequential action of three enzymes: Ube1L was used as the E1 enzyme, UbcH8 was used as the E2 enzyme, and HERC5 was used as the E3 enzyme. First, ISG15 binds the catalytically active cysteine of the Ube1L activating enzyme (E1) in an ATP-dependent reaction. Then, E1 interacts with the UbcH8 conjugating enzyme (E2) through its ubiquitin folding domain (UFD), which facilitates the transesterification of active ISG15 and results in an intermediate ISG15–UbcH8 complex joined by a thioester bond.^5^ Finally, the HERC5 ligase enzyme (E3) interacts with the intermediate ISG15–UbcH8 complex to mediate the ligation of ISG15 to the target protein. UbcH8 plays a central role in ISGylation as it interacts with both E1 and E3 enzymes—making it a key target for the regulation of the ISGylation pathway.^6^

Under reducing conditions, E2 enzymes can spontaneously form dimers when a cross-linker is added,^7^ and apart from a few exceptions, E2 enzymes are capable of preserving their dimer form.^8,9^ Both the dimer and the monomer forms of E2 enzymes are capable of recruiting E3 enzymes and conjugating ubiquitin.^10^ Although dimeric E2 enzymes are perceived as more advantageous because one of the monomers can remain associated, the ubiquitin conjugation continues with the other.

The Protein Data Bank (PDB) contains both dimeric (PDB ID:1WZV) and monomeric (PDB ID:1WZW) crystal structures of UbcH8 (Figure S1^1^). However, there are no primary citation articles for either structure except for the PDB. It remains unknown whether UbcH8 dimerizes naturally or as a consequence of nonspecific crystal packing contacts. In this study, we aimed to characterize the oligomeric state of human UbcH8 (Ube2L6) in solution using small-angle X-ray scattering (SAXS). We first used a fusion protein approach with the goal of producing a high-resolution scattering envelope to properly place the UbcH8 protomers. Surprisingly, upon removal of the N-terminal fusion, the monomer–dimer equilibrium further shifted to the dimer side. We next used solution nuclear magnetic resonance (NMR) spectroscopy to validate and further characterize the monomer–dimer equilibrium. Our results indicate that UbcH8 contains a substantial dimer population at a concentration of 300 μM and that dimerization may induce conformational changes at the distal E1 and ISG15/E3 interaction interfaces.

**Figure 1:**
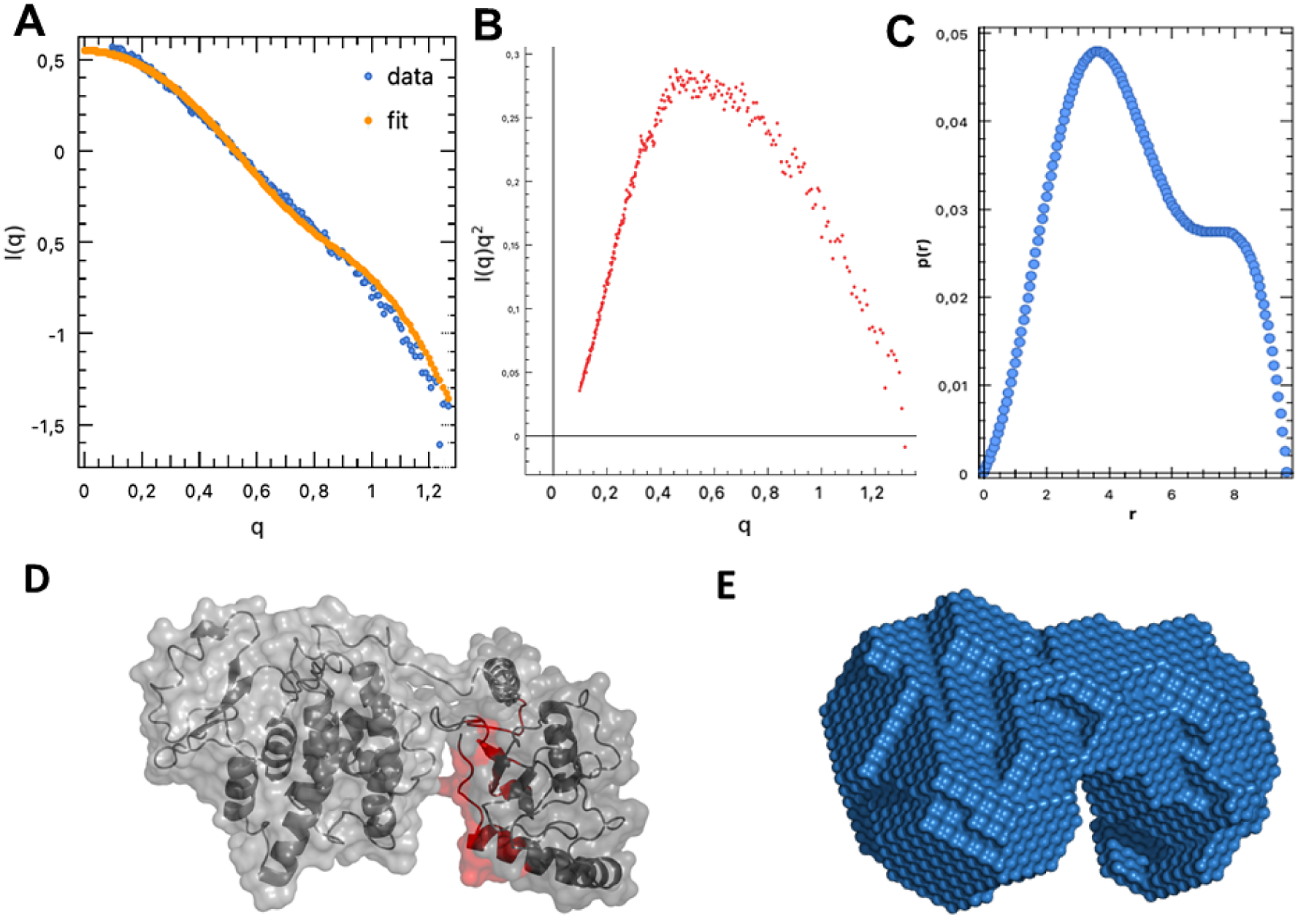
SAXS analysis of GST-UbcH8 fusion protein. (A) ln *I*(q*)* vs *q* plot, (B) Kratky plot, and (C) oair distance distribution, *P*(*r*), plot of the experimental SAXS intensity obtained at 4.1 mg/mL (300 μM) of the GST-UbcH8 fusion protein. The pair-distance distribution plot of the GST-UbcH8 fusion protein scattering data was calculated by GNOM. (D) The individual crystal structures of GST (left) (PDB: 1R5A) and UbcH8 (right (PDB: 1WZW) shown as cartoon representations; UbcH8 homodimeric dimerization surface labeled in red. (E) The GST-UbcH8 dummy atom model, obtained by the ATSAS online package, is shown as a surface representation.

## Results

### GST Fusion Guides SAXS Protein Structural Modeling

To determine the state of UbcH8 in solution, we expressed and purified it fused to an N-terminal glutathione-S-transferase (GST) tag, herein referred to as GST-UbcH8. We hypothesized that the 28 kDa GST molecule would be easily discernible from the smaller (18 kDa) UbcH8, and would dramatically improve the fit of SAXS scattering data to the structural model. The sample was concentrated to 300 μM, and six 10-min SAXS frames were collected over a total of 1 h. The superimposition of all 10-min frames confirmed that the X-ray beam produced little to no detectable radiation damage (Figure S2^2^). The medium-to-high *q* region, emphasized in the *q* versus *l*(*q*) plot, is consistent with a folded sample (Figure 1A). The Kratky plot possessed a bell-shaped curve that approaches zero after reaching a maximum at ∼3 sRg; this result is consistent with a properly folded globular protein (Figure 1B). While slight deviations between the typical Kratky plot and the dimensionless Kratky plot can aid in the assessment of flexibility, no apparent differences were observed.

**Figure 2:**
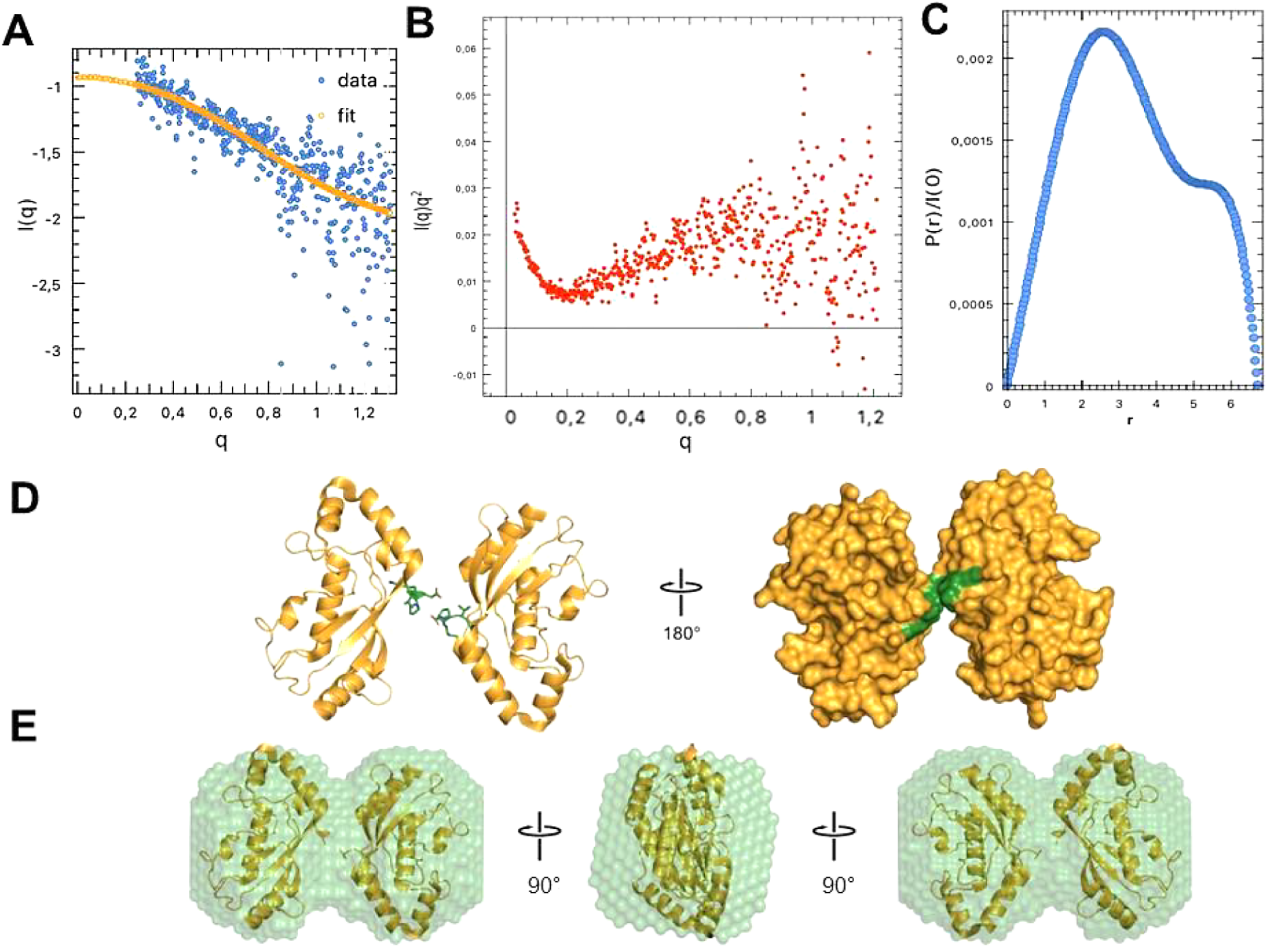
SAXS analysis of free UbcH8. (A) ln *I*(*q*) vs *q* plot, (B) Kratky plot, and (C) pair distance distribution, *P*(*r*), plot of the experimental SAXS intensity obtained at 4.1 mg/mL (300 μM) free UbcH8 protein. (D) The crystal structure of the UbcH8 dimer (pdb: 1WZV) shown as cartoon and surface representation using Pymol. Residues D149, R150, and P151 of the dimerization interface are colored forest green. (E) UbcH8 dimer crystal structure (pdb: 1WZV) fitted (χ = 1.02) into the DAMMIF dummy atom model using SASpy.

The pair-distance distribution function, *P*(*r*), is a measure of the frequency of interatomic distances that can also provide information about protein shape. The presence of a shoulder in the *P*(*r*) suggests a multidomain protein as expected for the GST-UbcH8 fusion (Figure 1C). The largest distance (*D*_max_) in the *P*(*r*) histogram was 8 nm (Figure 1C). The GST-UbcH8 crystal structures were then fitted into the final 3D DAMMIF dummy atom model (Figure 1D,E). In addition, molecular size parameters obtained with Primus are included in Table S1 and match the theoretically expected dimensions.

Both GST and UbcH8 proteins, as well as the linker peptide, are clearly visible, fitting a monomeric model. The fact that even the linker region can be detected and observed via SAXS analysis clearly underscores the power of SAXS in structure determination. After GST cleavage, we recorded unexpected UbcH8 size exclusion chromatographs. The UbcH8 peak was dispersed over a much wider elution volume compared to a similarly sized (12.2 kDa) monomeric protein injected under identical run parameters (Figure S3^3^), leading us to hypothesize that GST may block the dimerization site. Figure 1D shows the GST-UbcH8 fusion surface representation using the SWISS-MODEL; the UbcH8 homodimerization surface is shown in red. Both the SWISS-MODEL and the SAXS model positioned the GST fusion protein to occlude the UbcH8 dimerization interface.

**Figure 3:**
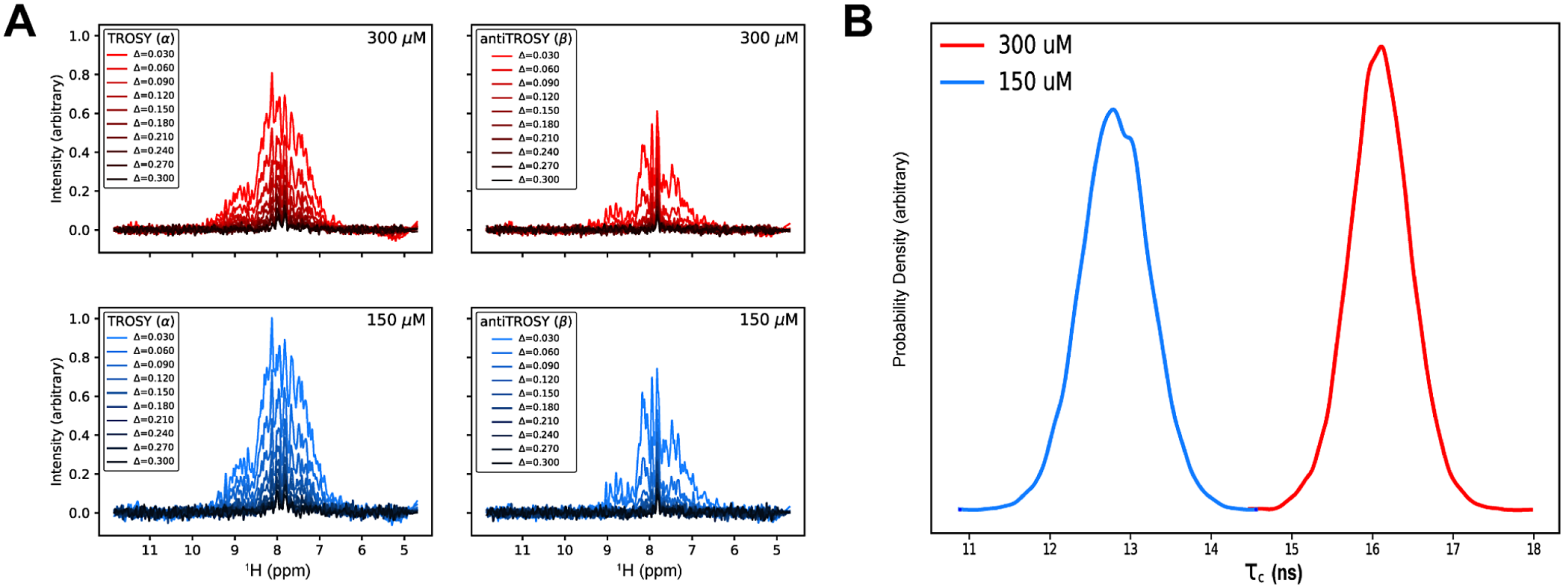
TRACT analysis of free UbcH8 to determine the rotational correlation time. (A) The UbcH8 1D ^15^N TROSY (left) and anti-TROSY (right) spectra from the TRACT experiment. The top and bottom panels are UbcH8 at 300 μM (red) and 150 μM (blue), respectively. We integrate from 8.6 to 9.2 ppm (gray boxed region) under the assumption that it possesses resonances from primarily structured regions. (B) The probability density estimates of the overall rotational correlation time (*τ*_*c*_) for UbcH8 at 300 μM (red) and 150 μM (blue). The average (point) estimate for 300 and 150 μM UbcH8 are 16.1 and 12.8 ns, respectively.

### FreeUbcH8 is a Dimer in Solution

To test how well GST-fusion improves the modeling of UbcH8 in SAXS scattering, we prepared a second UbcH8 sample with the GST protein removed. Again, we concentrated UbcH8 to 300 μM and collected six 10-min frames for a total acquisition time of 1 h (Figure 2A). Similar to GST-UbcH8, the Kratky plot possessed a bell-shaped curve approaching zero (Figure 2B). We estimated a slightly larger *R*_g_ ∼ 4.40 nm, compared to GST-UbcH8, from the low *q* region, whereas the *P*(*r*) *D*_max_ was reduced to 6.2 nm (Figure 2C). Surprisingly, the free UbcH8 *P*(*r*) also exhibited a shoulder, suggesting homodimerization (Figure 2C). ATSAS molecular weight analysis predicts a 39.5 kDa particle, which is approximately double the expected 18 kDa UbcH8. We then fitted the UbcH8 dimer crystal structure (PDB entry 1WZV; Figure 2D) to the dummy atom model of the scattering envelope (Figure 2E). The best-fit model (χ^2^ = 1.7) possesses a dimerization interface where the active site cysteines (Cys85) of each protomer are pointed outward (Figure 2D,E). The consistency between the previously published dimer crystal structure and the dummy atom model obtained by SAXS analysis supports UbcH8 homodimerization in the absence of a GST-tag.

### NMR Analysis of UbcH8 Monomer–Dimer Equilibrium

To further establish the dimerization of UbcH8 in solution, we performed Transverse Relaxation Optimized Spectroscopy (TROSY) for rotational correlation times (TRACT) experiments^11,12^ to estimate the rotational correlation time (*τ*_*c*_) of UbcH8 at two different concentrations: 300 and 150 μM (Figure 3). The signal intensity ranging from 8.6 to 9.2 ppm was integrated to maximize the signal-to-noise ratio and emphasize well-structured regions of the protein that are representative of global tumbling. We estimated the ^15^N relaxation rates for the TROSY and anti-TROSY integrated signals using Bayesian Parameter Estimation of a two-parameter single-exponential decay model. This method produces a distribution of decay rates that encompassess uncertainty, which was then used to determine the cross-correlated relaxation (CCR) rate. The rotational correlation time was estimated from CCR according to an algebraic solution^12^ of the modified Goldman relation^13^ assuming an order parameter (*O*^2^) of 0.8. We determined *τ*_*c*_ ∼ 16 ns at 300 μM and ∼13 ns at 150 μM (Figure 3), which demonstrates a concentration dependence on molecular rotation diffusion times. We then used hydroNMR^14^ to model the rotational diffusion of monomeric and dimeric UbcH8 based on the PDB 1WZV dimeric crystal structure. hydroNMR reported *τ*_*c*_ = 20.5 ns for the dimer and 7.4 ns for the monomer at 25 °C. Taken together, this confirms that UbcH8 undergoes monomer–dimer exchange and indicates a significant dimer population even at 150 μM. Data could not be collected at lower concentrations due to the sensitivity limit of the room temperature NMR probe.

**Figure 4:**
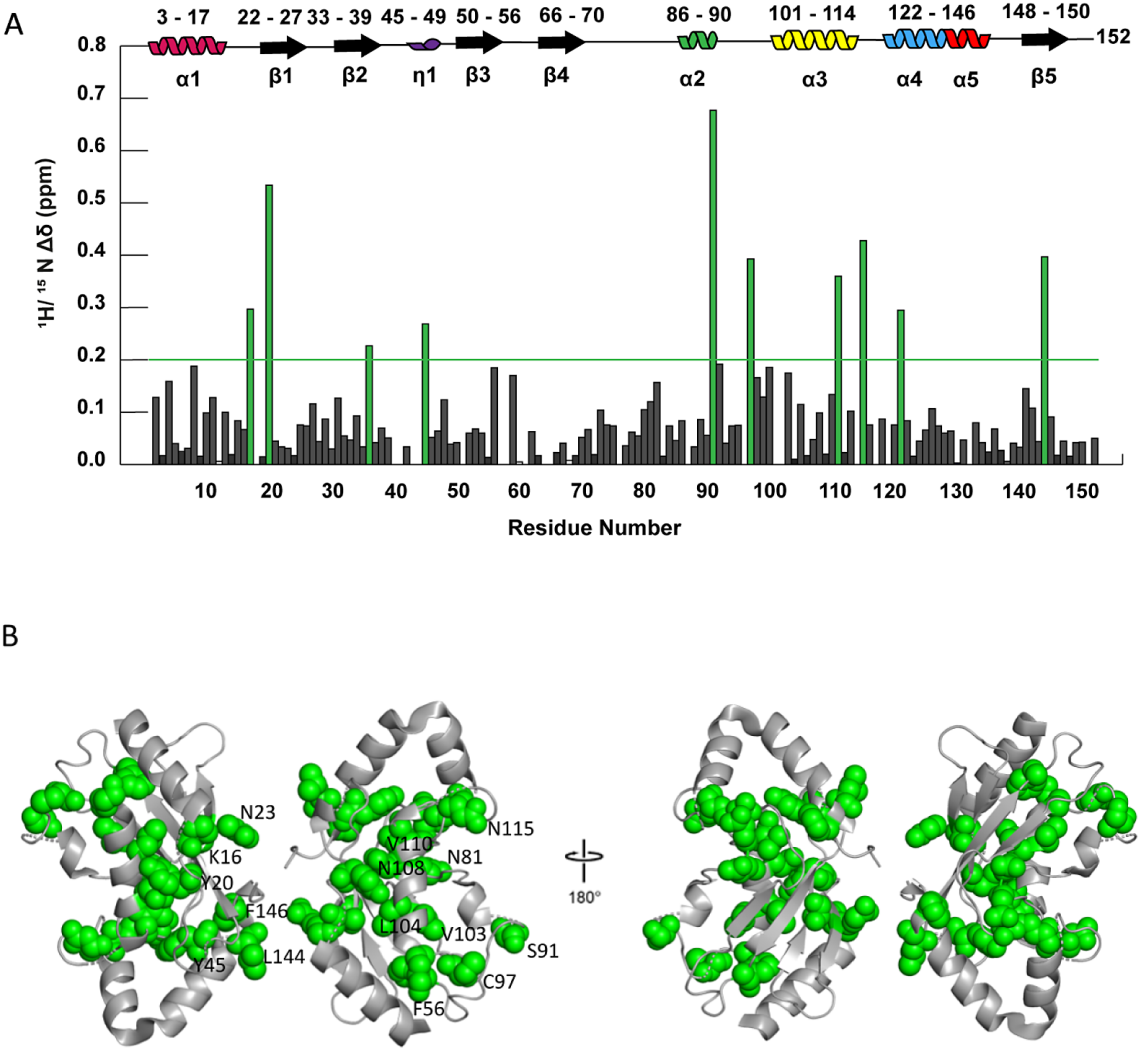
^1^H–^15^N chemical shift perturbations were mapped onto the UbcH8 dimeric crystal structure. (A) The combined ^1^H–^15^N chemical shift perturbations between 300 and 150 μM UbcH8 were calculated for each residue. The residues colored green possessed chemical shift perturbations larger than the threshold (green line). Residues with no bars were unobservable at either concentration. (B) Residues with chemical shift perturbations larger than the threshold were mapped onto the UbcH8 dimeric crystal structure (PDB 1WZV). These residues cluster to two distinct regions: the E1 binding surface (K17, Y21, A36, Y45) and the ISG15/E3 binding surface (S97, T100, V103, V111, and N115).

We then collected ^15^N heteronuclear single quantum coherence (HSQC) solution NMR spectra at 150 μM (Figure S4^4^) and 300 μM to identify UbcH8’s dimerization interface. Resonances were assigned by visual inspection using BMRB Entry ID 16321 as a reference list. NH resonances of all residues except for the 18 prolines were assigned (86.18% completion). We subsequently assessed concentration-dependent chemical shift perturbations (CSPs) and peak intensity differences. In Figure 4A, we observe significant chemical shift changes for the E1 binding surface (K17, Y21, A36, and Y45) and the ISG15/E3 binding surface (S97, T100, V103, V111, and N115). Substantial concentration-dependent CSPs were induced at locations far from the crystallographic dimerization interface (Figure 4B). All of the perturbed residues, except for K17 and Y21, are situated at either the E1 or the ISG15/E3 interaction surfaces. Residues S91, E92, and S97 are clustered on a loop near the catalytic C85 residue, where ISG15 is covalently attached. Additionally, S97, T100, V103, V111, and N115 are proximal to the ISG15/E3 binding region on the UbcH8 surface; interestingly, these residues are arranged toward the UbcH8 core rather than at the surface (Figure 4B). Given that E1 and ISG15/E3 involve distinct interfaces, we hypothesize that a conformational change or allosteric pathway influences the transfer or binding of ISG15. Our results suggest that dimerization may play an additional role in ISGylation. We hypothesize that the weak CSPs could reflect a mostly side chain-mediated interface and/or that the ensemble is predominantly dimeric even at 150 μM concentration.

**Figure 5:**
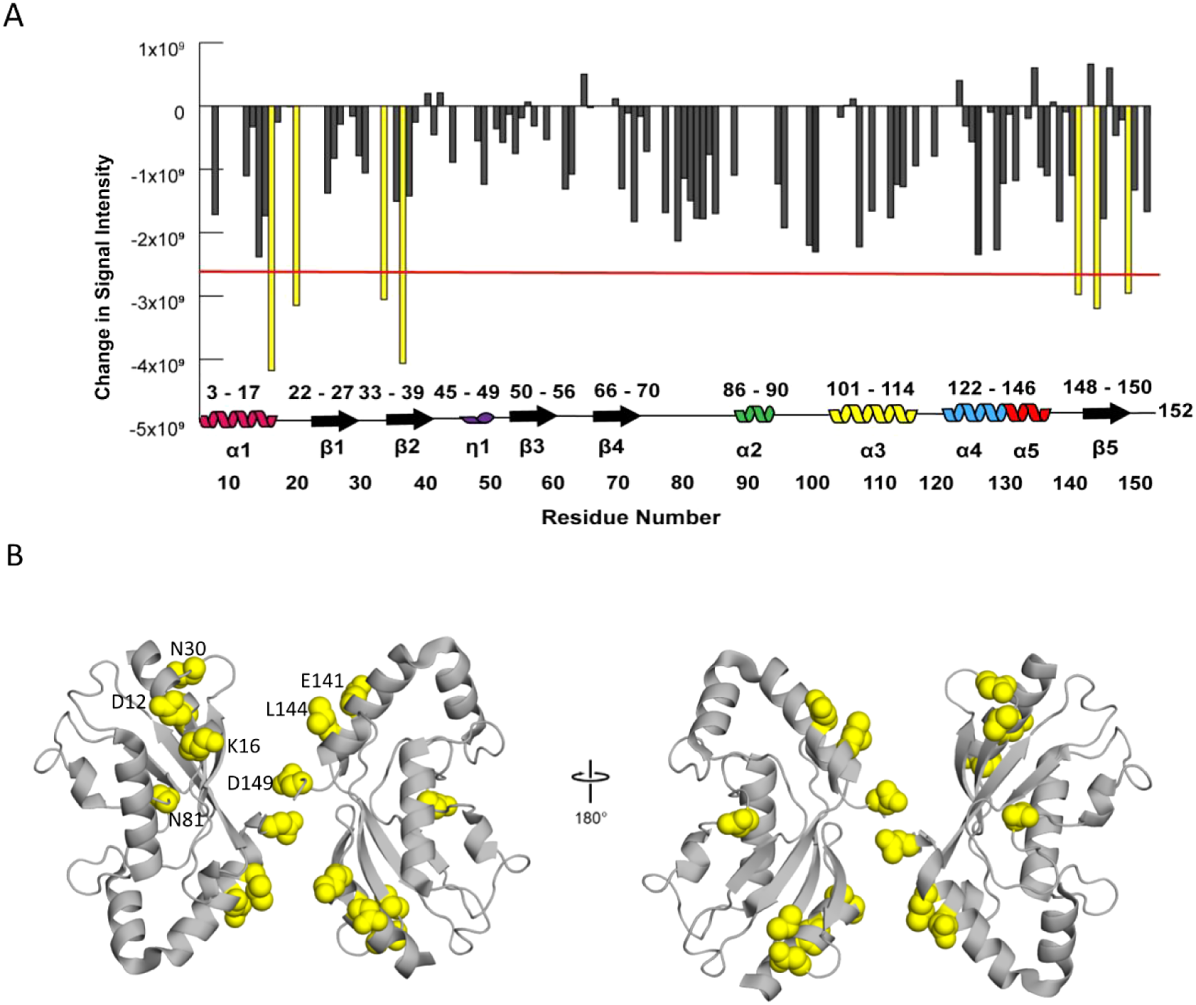
Concentration-dependent chemical shift signal intensity changes are depicted by: (A) Concentration-dependent changes in ^1^H–^15^N peak intensity mapped onto the UbcH8 dimeric crystal structure. The yellow residues possessed peak intensity changes that were larger than the threshold (red line). These residues are located at the E1 binding surface (D12, K16, N30, and V33) and C-terminal (E141, L144, and D149). Residues with no bars were not observed at either concentration. (B) Residues with peak intensity changes larger than the threshold were mapped onto the UbcH8 dimeric crystal structure (PDB entry 1WZV).

Thus, we also measured the concentration-dependent changes in the peak intensity. We hypothesize that these intensity differences result from a monomer–dimer exchange on the intermediate (microsecond–millisecond) time scale, but it is also possible that they reflect dimerization-dependent fluctuations in longitudinal (T_1_) or transverse (T_2_) relaxation. The largest changes in peak intensity again clustered to the E1 and ISG15/E3 interfaces while also highlighting an extended region along the crystallized dimer interface (Figure 5). D149 sits at the center of the dimerization surface with E141 and L144 in close proximity. It is plausible that D28 and A29, located in a loop region of the opposing protomer, could possess the flexibility to interact. Furthermore, as the overall structure gets bigger with the dimerization, decreased signals from some peaks were expected due to line broadening. Unlike the CSP analysis, which showed that most of the conformational changes occurred away from the dimerization site, the delta chemical shift intensity analysis revealed that most of the affected residues were on the dimerization site. In fact, the peak intensity of N23 and D149 residues from opposing protomers, which are within a 3.3 Å distance in the crystal structure, deviated from the mean peak height by 44.7% and 62.2% at 150 μM, and 37.6% and 35.6% at 300 μM, respectively (Figure S5^5^). Taken together, this indicates that dimerization is *ipso facto* involved in defining the interaction dynamics between E1 and E2 enzymes.

As a further attempt to investigate the UbcH8 dimerization effect on noncovalent interactions between ISG15 and UbcH8, 260 μM ^15^N-labeled UbcH8 was titrated with unlabeled ISG15. The chemical shift perturbations were mapped onto the UbcH8 structure and observed to affect different residues (Figure S6) compared to the 150–300 μM UbcH8 spectra. The catalytic C85 residue, to which ISG15 binds, is located on the opposite side of the proposed dimerization site on UbcH8. This binding surface is similar to the ubiquitin-binding surface on UbcH8.^15^ In addition, since we only examined noncovalent interactions, the changes in our CSP values are lower than the values obtained by covalently binding ubiquitin to UbcH8 with a disulfide bond.^15^ Based on the data, rather than dimerization inhibiting ISG15 interaction, it is more likely that dimerization may affect E1 and/or E3 interactions, thus negatively regulating the ISG15 conjugation pathway.

## Discussion

In this study, we investigated the oligomeric state of UbcH8 in solution by using small angle X-ray scattering (SAXS) and NMR analysis. To improve the fitting of SAXS scattering data to the structural model, we initially employed an N-terminal GST-fused UbcH8 protein. The results from SAXS experiments and analysis indicate that the GST-UbcH8 fusion protein is monodisperse and properly folded in solution. The 3D DAMMIF dummy atom model also revealed a monomeric model of the GST-UbcH8 fusion protein, highlighting the advantages of SAXS in structure determination. The free form of UbcH8, without GST fusion, was further investigated. Surprisingly, the *P*(*r*) distribution suggested a multidomain complex, and the ATSAS molecular weight analysis indicated dimer formation. The consistency between the existing crystal structure of the UbcH8 dimer and the SAXS-derived dummy atom model supports the formation of a UbcH8 homodimer in solution in the absence of a GST-tag.

Our NMR analysis, including chemical shift perturbation and peak intensity measurements, provides additional evidence for the dimerization of UbcH8. Residues involved in the dimerization process were identified, and the effects of dimerization on the E1 and E3 interaction sites were observed. The TRACT experiments also supported the dimerization of UbcH8, revealing a concentration-dependent behavior of UbcH8 in solution, which suggests monomer–dimer exchange on an intermediate time scale. The dimerization of UbcH8 and its implications on the ISGylation process are consistent with previous reports of E2 enzymes forming dimers to facilitate polyubiquitination.^10^ Dimerization of several E2 enzymes has been reported, and studies have shown that this observed dimerization of the enzymes is found to stimulate the catalytic activity of the E2 enzyme.^9,16−18^ In 2010, David et al. suggested that E2 enzymes form dimers in solution regardless of the presence of active ubiquitin.^7^ While the monomeric form is also active for the acquisition of ubiquitin, the dimeric form of the E2 is found to be more advantageous because one monomer site can bind ubiquitin molecules, while the other site is capable of remaining associated with the target protein, facilitating efficient polyubiquitination. The acting mechanism of E2 enzymes proposed in this study suggests that the E2 enzymes function as dimers while catalyzing the polyubiquitination process.^7^ An example of dimerization is observed for the E2 enzyme UBE2W.^8^ An equilibrium between the monomeric and dimeric states was observed and verified using biochemical assays, as well as NMR experiments. To compare the type of UbcH8 dimerization with previous dimer E2 structures, we screened the fitting of free UbcH8 dummy atom model with previously determined dimeric E2 enzyme crystal structures (Figure S7 and Table S2). These structural screening results showed us that the dimerization pattern of UbcH8 is most similar to that of UbcA1 from the Ube2w family (Figure S8).

In another study conducted on the Ub E2 enzyme UBE2S, intracellular colocalization was observed and the presence of oligomeric states was measured using NMR.^9^ Additionally, the authors propose a dimerization model where the C-terminal helices of the protein monomers “hug” the other, generating a dimer that is not auto-ubiquitinated, as opposed to the monomeric form. Preventing auto-ubiquitination allows UBE2S to exist at higher intracellular concentrations without being degraded by the proteasome, even though its ubiquitination activity is impaired. This mechanism shows how dimerization could be relevant for the regulation of ubiquitination activity. As reviewed by ref. (6), the ubiquitination activity of certain E2s can also be modulated by the binding of Ub to a secondary site known as the backside, distinct from the catalytic surface. For different E2 enzymes, the poly Ub chain formation capability can be enhanced or impaired by the binding of Ub on the backside surface. Additionally, the reported binding of E3 enzymes on the backside^6^ also raises the question of how ubiquitination activity is regulated by the equilibrium between E1–E2 and E2–E3 interactions and how E2 dimerization could affect this process. The complex interaction dynamics of E2 with itself, the conjugated protein, and the E3 enzymes have yet to be revealed, including how dimerization affects this regulation. Dimerization could reduce E2 activity, as seen in the case of UBE2W, by limiting backside accessibility to E3 or the conjugate Ubl protein. Conversely, the E1 and E3 interaction surfaces on the backside might be exposed more, leading to increased E2 activity. Overall, E2 dimerization holds significance for regulatory mechanisms that have yet to be fully revealed.

ISG15 and related enzymes are expressed at elevated levels when an infection is present or when stimulated by IFNs. SARS-CoV-2-infected macrophages were reported to have more than a 100-fold increase in ISG15 mRNA levels, with the measured intracellular protein concentration reaching >700 ng/mL (∼0.5 μM in nucleus).^19^ Similarly, mRNA transcript levels of the E1 and E3 enzymes, Ube1L and HERC5, increased to 4 and 40 times the basal level during SARS-CoV-2 infection, respectively. An 8-fold increase for UbcH8 transcripts was also observed under the same conditions.^19^ Data regarding the molar concentration of UbcH8 are not available; however, single-cell RNA data for macrophages report 64.8 nTPM (normalized transcript per million) and 138.4 nTPM for UbcH8 and ISG15, respectively.^20−23^ Hence, we consider that the intracellular UbcH8 concentration might be close to that of ISG15.

Considering that the CSP data indicate a possible conformational change in the E1 and E3 binding sites on UbcH8 associated with dimerization, it is also possible that elevated levels of E1 and E3 within the cell promote the dimeric state, thereby increasing E2 activity. Our results demonstrate that UbcH8, the E2 enzyme specific for ISGylation, may also form dimers at near physiological concentrations due to the effects of molecular crowding and excluded volume effect. Although our experiments were performed at high concentrations, we believe that UbcH8 dimerization can also occur at lower concentrations within cells due to the effects of crowding and excluded volume. The crowded cellular environment effectively increases the local concentration of UbcH8 molecules, bringing them to closer proximity and enhancing the likelihood of interactions that can lead to dimerization. Additionally, the excluded volume effect limits the available space for UbcH8 molecules, further promoting interactions and potentially favoring dimer formation over other conformations. Locally high concentrations of proteins can occur due to macromolecular crowding, which can make protein–protein complex formation reactions more thermodynamically favorable.^24^ This effect can modulate the behavior of proteins in the intracellular environment, where numerous macromolecules are present, and it provides suitable conditions for oligomer formation at low molar concentrations.

This suggests that the ISGylation process may also involve dimerization to regulate the complicated interactions of E1, E2, and E3 enzymes. This study highlights the importance of understanding the oligomeric state and behavior of proteins in solution to gain insight into their biological function and regulation. Moreover, our work emphasizes the usefulness of SAXS and NMR techniques in elucidating protein structures and interactions in solution, which can complement crystallographic studies and provide a more biologically relevant context.

Future studies may focus on exploring the functional implications of UbcH8 dimerization in the ISGylation process, such as its effects on substrate specificity, E1 and E3 enzyme interactions, and the kinetics of ISGylation. Additionally, the molecular mechanisms underlying the observed concentration-dependent behavior of UbcH8 and the role of post-translational modifications in modulating its oligomeric state could be investigated further. These studies will contribute to a more comprehensive understanding of the regulation and function of UbcH8 in the context of ISGylation and its broader implications in various diseases, including viral and bacterial infections, cancer, and autoimmune disorders.

## Materials and Methods

### Protein Expression and Purification

Three alanine residues followed by the coding sequence of the UbcH8 protein are inserted in the 5′ BamH1/3′ EcoR1 restriction sites of the pGEX-4T3 plasmid. Three alanine residues are inserted between the GST and UbcH8 protein sequences in order to increase the binding efficiency and provide better cleavage upon thrombin treatment during the purification of the protein sample. The final coded amino acid sequence was: MSPILGYWKIKGLVQPTRLLLEYLEEKYEEHLYERDEGDKWRNKKFELMGLEFPNLPYYIDGDVKLTQSMAIIRYIADKHNMLGGCPKERAEISMLEGAVLDIRYGVSRIAYSKDFETLKVDFLSKLPEMLKMFEDRLCHKTYLNGDHVTHPDFMLYDALDVVLYMDPMCLDAFPKLVCFKKRIEAIPQIDKYLKSSKYIAWPLQGWQAFGGGDHPPKSDLVPRGSAAAMASMRVVKELEDLQKKPPPYLRNLSSDDANVLVWHALLLPDQPPYHLKAFNLRISFPPEYPFKPPMIKFTTKIYHPNVDENGQICLPIISSENWKPCTKTCQVLEALNVLVNRPNIREPLRMDLADLLTQNPELFRKNAEEFTLRFGVDRPS*.

pGEX-4T3 GST-AAA-UbcH8 plasmid was transformed into Rosetta2 *E. coli* expression cells, plated on LB–ampicillin–chloramphenicol, and grown overnight at 37 °C. The next morning, colonies were picked from the agar plate and inoculated into 10 mL LB–ampicillin–chloramphenicol medium. The culture was grown overnight at 37 °C and 110 rpm. The overnight culture was transferred into 1 L LB medium and incubated at 37 °C. After OD_595_ exceeded 0.3, temperature was lowered to 18 °C and protein production was induced at OD_595_ 0.8 by the addition of 0.4 mM IPTG. Cells were harvested 18 h after induction by centrifugation at 2000 RCF for 1 h.

Harvested cells were resuspended in lysis buffer (500 mM NaCl, 50 mM Tris, 0.1% (v/v) Triton X-100, 5% (v/v) glycerol, 1 mM DTT, pH 7.5), sonicated, and centrifuged at 20K RCF for 1 h to remove insoluble debris. The obtained supernatant was loaded onto a GST affinity column equilibrated with 20 mM Tris (pH 7.5), 150 mM NaCl, and 1 mM DTT. Nonspecific proteins were washed with the same buffer, and the protein was eluted with 30 mM glutathione, 20 mM Tris (pH 7.5), 150 mM NaCl, and 1 mM DTT. For cleavage of the GST tag, thrombin enzyme (1:100) was added to the eluted protein and dialyzed in 20 mM Tris (pH 7.5), 150 mM NaCl, 1 mM DTT solution overnight to eliminate excess glutathione. For the separation of the GST tag, reverse GST chromatography was applied. Unbound free UbcH8 was collected and purified by size exclusion chromatography using 20 mM Tris (pH 7.5), 150 mM NaCl, and 1 mM DTT buffer. Unlabeled ISG15N13Y/C78S protein was also expressed and purified as described above for NMR titration studies.

### SAXS Data Collection

All SAXS data were collected at home source SAXSpoint 5.0 (Anton Paar GmbH) as described before.^25^ The sample/detector distance (SDD) was 1600 mm for SAXS experiments. All measurements were conducted at 10 °C. Data were collected in 1 h sessions (1 min long 6 frames) for each measurement. The scattering curves were checked for radiation damage, and no damage was detected after superimposing each 10-min data collection interval.

### SAXS Data Processing and Modeling

First, the scattering pattern of all samples were visually inspected in the Primus program of ATSAS 3.0 for any possible measurement issues.^11^ The radius of gyration (*R*_*g*_) was calculated using Guinier’s equation and inverse Fourier transform by Primus. The distance distribution function *P*(*r*) and the maximum particle diameter (*D*_max_) were calculated by GNOM.^26^ After estimating the molecular weight of the model, DAMMIF (ab initio) was used to generate five independent low resolution models from the data.^27^ DAMAVER and DAMMIN then averaged, clustered, and optimized these five distinct solutions to form the final ab initio shape.^28^ The SASpy plug-in for PyMOL was used to superimpose the homology-modeled structure of the protein.^29,30^

### ^15^N-Labeled Protein Expression and Purification

pGEX-4T3 GST-AAA-UbcH8 plasmid containing bacteria were grown overnight in LB medium at 37 °C and transferred into 50 mL of ^15^N-labeled M9 media the next day. Following 4 h of incubation at 37 °C, the cells were transferred into 1L M9 media. After the OD_595_ exceeded 0.3, the temperature was lowered to 18 °C, and protein production was induced at an OD_595_ of 0.8 by the addition of 0.4 mM IPTG. The medium contained 33.7 mM Na_2_HPO_4_, 22 mM KH_2_PO_4_, 8.55 mM NaCl, 9.35 mM ^15^N-labeled NH_4_Cl, 1 mM MgCl_2_, 0.3 mM CaCl_2_, and 7 mg/L FeCl_2_-4H_2_O. The cells were harvested 18 h after induction by centrifugation at 2000 RCF for 1 h.

The harvested cells were resuspended in lysis buffer (500 mM NaCl, 50 mM Tris, 0.1% (v/v) Triton X-100, 5% (v/v) glycerol, 1 mM DTT, pH 7.5), sonicated, and centrifuged at 20K RCF for 1 h to remove insoluble debris. The obtained supernatant was loaded onto a GST affinity column equilibrated with 38.39 mM Na_2_HPO_4_, 11.61 mM KH_2_PO_4_ (pH 7.4), 100 mM NaCl, and 1 mM DTT. Nonspecific proteins were washed with the same buffer and the protein was eluted with 30 mM glutathione, 38.39 mM Na_2_HPO_4_, 11.61 mM KH_2_PO_4_ (pH 7.4), 100 mM NaCl, and 1 mM DTT. For cleavage of the GST tag, thrombin enzyme at a ratio of 1:100 was added to the eluted protein and dialyzed in 38.39 mM Na_2_HPO_4_, 11.61 mM KH_2_PO_4_ (pH 7.4), 100 mM NaCl, and 1 mM DTT solution overnight to eliminate excess glutathione. For separation of the GST tag, reverse GST chromatography was applied. Unbound free UbcH8 was collected and purified by size exclusion chromatography using 38.39 mM Na_2_HPO_4_, 11.61 mM KH_2_PO_4_ (pH 7.4), 100 mM NaCl, and 1 mM DTT buffer.

### NMR Data Acquisition and Analysis

UbcH8 was concentrated to 0.150 and 0.287 mM. A final concentration of 10% D2O containing 1 mM DSS was added to obtain a final sample volume of 600 μL. For NMR titration, UbcH8 was concentrated to 0.300 mM, and a series of 1H, ^15^N-HSQC spectra were acquired from samples with ^15^N-UbcH8 and unlabeled ISG15 at molar ratios of 1:0.25, 1:0.5, 1:1, 1:2, and 1:4. All NMR data acquisition process was completed using a 500 MHz Bruker Ascend magnet equipped with an Avance NEO console and a BBO double resonance room temperature probe at the Koç University n^2^STAR NMR Facility. 2D ^1^H–^15^N HSQC spectra were recorded with 50% nonuniform sampling (NUS) at 298 K with a 1H spectral width of 14 ppm (1024 data points in t2) and a ^15^N spectral width of 32 ppm (64 data points in t1). The 2D data were processed by NMRPipe^31^ and analyzed using NMRFAM-SPARKY.^32^ The combined ^1^H–^15^N chemical shift perturbations were calculated using equation ΔδAV = [(Δδ 1H * 5)^2^ + (Δδ 15N)^2^]^1/2^.

1D TRACT experiments^11^ were collected with 1024 complex points and a 1.5 s recycle delay. Relaxation rates for ^15^N TROSY and anti-TROSY components were determined from spectra intensity values integrated over 9.2 to 8.6 ppm at eight relaxation delays: 30, 60, 90, 120, 150, 180, 210, 240, 270, and 300 ms. Each relaxation rate and its uncertainty were estimated by fitting the integrated values and time delays to a single parameter exponential decay model using Bayesian parameter estimation. Each TROSY and anti-TROSY relaxation rate was then used to estimate the rotational correlation time (*τ*_*c*_) using the algebraic method,^12^ where we assumed an order parameter (*O*^2^) of 0.8.

## Supporting Information Available

Additional SAXS data analysis tables, superimposition of SAXS data, ^1^H–^15^N HSQCs, and dummy fittings (PDF)
